# Potential role of exosome-associated microRNA panels and *in vivo* environment to predict drug resistance for patients with multiple myeloma

**DOI:** 10.18632/oncotarget.9021

**Published:** 2016-04-26

**Authors:** Li Zhang, Ling Pan, Bing Xiang, Huanling Zhu, Yu Wu, Meng Chen, Pujun Guan, Xingli Zou, C Alexander Valencia, Biao Dong, Jianjun Li, Liping Xie, Hongbing Ma, Fangfang Wang, Tian Dong, Xiao Shuai, Ting Niu, Ting Liu

**Affiliations:** ^1^ Department of Hematology, West China Hospital, Sichuan University, Chengdu, China; ^2^ Department of Medical Oncology, Dana-Farber Cancer Institute, Boston, Massachusetts, USA; ^3^ Department of Radiology, West China Hospital, Sichuan University, Chengdu, China; ^4^ Department of Hematology, Affiliated Hospital of North Sichuan Medical College, Nanchang, China; ^5^ Division of Human Genetics, Cincinnati Children's Hospital Medical Center and Department of Pediatrics, University of Cincinnati College of Medicine, Cincinnati, Ohio, USA; ^6^ State Key Laboratory of Biotherapy, West China Hospital, Sichuan University, Chengdu, China

**Keywords:** myeloma, resistance, exosome, microvesicle, microRNA

## Abstract

Multiple myeloma (MM) is the second most common hematologic neoplasms and an appropriate *in vivo* environment for myeloma cells has potential implications for initiation, progression, and metastasis of MM. Exosomes, entities carrying microRNAs (miRNAs) to target locations, participate in the cross-talk between myeloma cells and nonmalignant components of the *in vivo* environment. This study disclosed the emerging roles of circulating exosome-associated miRNAs in drug resistance (DR) of MM. To this end, the medical records of consecutively hospitalized MM patients, who received novel agents-based therapies, were analyzed. Then, an optimized procedure was established for exosome isolation and exosomal RNA analysis. The exosome-associated miRNA expression patterns for predicting bortezomib (Bz) resistance of MM were further examined using a microarray. In total, 204 patients were enrolled with DR rates of 36.5%, 73.1% and 81.8% in the bortezomib (Bz), thalidomide and lenalidomide containing groups. The serum total light chain ratio ≥ 100, CRP ≥ 20 mg/L, and the second-line usage increased risks of acquired Bz-resistance. Among 68 cases having genetic tests, a high risk factor for predicting *de novo* DR was 1q21 amplification, which also correlated with lower levels of cholesterol and LDL-C. Moreover, nano-sized exosomes were isolated with significantly increasing internal RNAs and down-regulation of exosomal miR-16-5p, miR-15a-5p and miR-20a-5p, miR-17-5p was revealed in the patients resistant to Bz. The routine workup of MM hardly suggested a value for DR prediction. The circulating exosomes carrying miRNAs provided a window that permits a better understanding of the *in vivo* intercellular crosstalk in MM patients.

## INTRODUCTION

Multiple myeloma (MM) is a common and life-threatening hematological malignancy and is characterized by uncontrolled growth and accumulation of monoclonal plasma cells. These cells can infiltrate the bone marrow (BM) and typically secrete monoclonal (M) proteins into the peripheral circulation, resulting in related organ and tissue injury (ROTI). The pathogenesis of MM is largely attributed to the interplay between myeloma cells and the *in vivo* environment, mostly the bone marrow microenvironment (BMME). This interaction occurs throughout the entire disease process, namely, from monoclonal gammopathy of undetermined significance (MGUS), smoldering MM, symptomatic MM, and finally to plasma cell leukemia (PCL) [1]. Conventional therapies such as hematopoietic stem cell transplantation, which mainly focused on myeloma cells, result in a low complete remission (CR) rate and short survival time in MM [2]. The emerging novel therapies, including proteasome inhibitors and immunomodulatory drugs (IMiDs), can influence the BMME of MM and have strikingly improved the survival for MM patients [3–5]. However, resistance to novel drugs tends to be a clinical frustration because nearly all MM patients inevitably relapse or evolve to a refractory stage. In addition to the biobehavioral changes of myeloma cells of resisting drug challenges, BMME such as bone marrow stromal cells (BMSCs), have been found to play a vital role in drug resistance like bortezomib [6]. Thus, more attention needs focused on the crosstalk between myeloma cells and the *in vivo* environment, which may shed light on understanding drug resistance in MM. Accordingly, it is necessary to find some routine clinical markers that reflect the *in vivo* environment directly correlated to a high predictive value of DR.

We designed this study using the real world study (RWS) method of using data for decision making that was not collected in randomized clinical trials (RCTs), a concept introduced in 2007 by the International Society for Pharmacoeconomics and Outcomes Research (ISPOR) Task Force [7]. Additionally, due to focal distribution of the disease it is difficult to obtain samples containing myeloma cells and those that are obtained may lack biomarkers that reflect MM [8]. Exosomes, which are released into circulation from all cell types, are lipid bilayer cup-shaped nanovesicles with 30–100 nm in diameter and provide membrane protection for inclusive RNAs and proteins [9]. MicroRNAs (miRNAs), existing naturally as the most biologically stable nucleic acid molecule with only about 19–23 nucleotides, act as fine-tuning regulators of gene expression at post-transcriptional level through a complicated miRNA-mRNA interaction [10]. Until now, emerging studies have suggested that tumor-derived exosomes quantitatively predominate in peripheral blood and exosome-mediated miRNA transduction plays a pivotal role in the dialogue between human tumors and their microenvironment [11]. Thereby, we hypothesized that the profile of exosomal miRNA from peripheral blood, which can be easily available with a minimally invasive procedure, had a predictive value of primary or acquired drug resistance (DR) for MM patients.

## RESULTS

### Patients' characteristics

A total of 300 MM patients with 1826 episodes of hospitalizations (with an average of 6.1 per patient per year) were analyzed in our center. The median age of this cohort was 61 years old, while the estimated median OS was 83.9 months. As shown in Table 1, 68.0% (*n* = 204) of patients with 1682 episodes of hospitalizations received a novel agent-based regimen and were enrolled in the study, among which, 56.4% (*n* = 115), 32.8% (*n* = 67) and 10.8% (*n* = 22) were treated with Bz, thalidomide and lenalidomide, respectively. Meanwhile, 32% (*n* = 96) of the remaining patients were admitted for clinical trials or supportive treatment. Bz and thalidomide were mainly used as front-line therapy, while lenalidomide as salvage therapy. In total, DR accounted for 53.4% (*n* = 109) of the entire cohort with the lowest frequency of 36.5% (*n* = 42) in the Bz group (*p* = 0.000).

**Table 1:** Baseline characteristics of the MM patient cohort receiving a novel agent-based regimen

Characteristics		All (*n* = 204)		Bz (*n* = 115)		Thalidomide (*n* = 67)		Lenalidomide (*n* = 22)		*p*
		*n*	%	*n*	%	*n*	%	*n*	%	
Age (year) (*n* = 204)	< 65 ≥ 65 Median age	143 61 61	70.1% 29.9%	90 25 60	78.3% 21.7%	40 27 62	59.7% 40.3%	13 9 62.5	59.1% 40.1%	0.015
Gender (n = 204)	M F	120 84	58.8% 41.2%	74 41	64.3% 35.7%	35 32	52.2% 47.8%	11 11	50.0% 50.0%	0.187
ISS stage (n = 204)	I II III Missing I II and III	40 60 51 53 40 111	26.5% 73.5%	20 38 32 25 20 70	22.2% 77.8%	18 19 13 17 18 32	36.0% 64.0%	2 3 6 11 2 9	18.2% 81.8%	0.169
D-S stage (n = 204)	1A 1B 2A 2B 3A 3B Missing 1 2 and 3 A B	18 0 61 7 61 26 31 18 155 140 33	9.9% 90.1% 83.8% 16.1%	6 0 37 2 37 15 18 6 91 80 17	6.2% 93.8% 82.5% 17.5%	11 0 20 4 18 9 5 11 51 49 13	17.7% 82.2% 79.0% 21.0%	1 0 4 1 6 2 8 1 13 11 3	7.1% 92.9% 78.6% 21.4%	0.061 0.842
Group (n = 204)	Response group DR De novo DR Acquired DR	95 109 88 21	46.6% 53.4% 43.1% 10.3%	73 42 35 7	63.5% 36.5% 30.5% 6.0%	18 49 40 9	26.9% 73.1% 59.7% 13.4%	4 18 13 5	18.2% 81.8% 59.1% 22.7%	0.000
Isotype of M protein (n = 204)	κ λ IgG κ IgG λ IgA κ IgA λ Nosecretory IgM λ IgG IgA λ Missing Light-chain Intact Ig Nosecretory	11 18 56 49 26 24 5 2 1 12 29 158 5	15.1% 82.3% 2.6%	9 13 30 22 10 17 2 2 1 9 22 82 2	20.8% 77.4% 1.9%	2 4 20 22 11 2 3 0 0 3 6 55 3	9.4% 85.9% 4.7%	0 1 6 5 5 5 0 0 0 0 1 21 0	4.5% 95.5% 0	0.092
Cytogenetics (n = 68)	Standard-risk High-risk	36 32	52.9% 47.1%	27 22	55.1% 44.9%	7 8	46. 7% 53.3%	2 2	50.0% 50.0%	0.843
Front-line therapy or not (n = 204)	Front-line therapy Second-line therapy	137 67	67.2% 32.8%	82 33	71.3% 28.7%	49 18	73.1% 26.9%	6 16	27.3% 72.7%	0.000
Survival (n = 204)	Estimated OS (months)	83.9 (77.8–90.0)		80.1 (74.2–87.0)		84.4 (73.2–95.5)		68.0 (58.4–78.1)		0.249

### Baseline data predicting DR for MM treated with novel agents

As shown in Tables 2–3, the *de novo* DR group accounted for 83.38% (*n* = 35) of the Bz-resistant group, while 73.13% (*n* = 40) of the thalidomide-resistant group. For the drug resistant cases receiving bortezomib-containing regimens, compared with the response, also called the sensitive group, the serum total light chain ratio was higher than 100 or less than 0.01 RR = 34.286; 95% CI: 3.476–338.2 *P* = 0.001), CRP was greater than 20 mg/L (RR = 14; 95% CI: 1.23–158.84 *P* = 0.032), and the second-line usage of bortezomib (RR = 9.667; 95%CI: 1.705–54.87; *P* = 0.009) was associated with an increased risk for the occurrence of acquired resistance. However, no significant indicators from the routine workup were found between the response and primary resistant groups capable of predicting the efficacy for the usage of either Bz or thalidomide as well as between the response and acquired thalidomide-resistant group.

**Table 2:** The relationship between the *in vivo* environment and treatment efficacy of bortezomib for MM

	Index	Response group (*n* = 73)		De novo DR (*n* = 35)		Acquired DR (*n* = 7)		*p*
		Mean	SD	Mean	SD	Mean	SD	
Age (year)	Median age and range ≥ 65 < 65	59 (36–74) 58 15		60 (28–80) 27 8		63 (46–72) 5 2		0.87
Treatment condition (*n* = 115)	Front-line therapy Second-line therapy	58 15		22 13		2 5		0.007
Patients' general information: (*n* = 115)	Isotype of M protein Intact Ig Light chain	53 17	75.7% 24.3%	22 5	81.5% 28.5%	7 0	100% 0	0.301
	Proportion of M protein	0.34	0.20	0.33	0.19	0.22	0.09	0.548
	Serum total involved/uninvolved light chain ratio 2–5:1 ≤ 0.01 or ≥ 100 0.01< ratio > 2 and 5 < ratio > 100	4 7 48	6.8% 11.9% 81.4%	5 1 21	18.5% 3.7% 77.8%	0 5 1	0 83.3% 16.7%	0.000
	Hb (13.0–17.5 g/dL)	99.2	24.2	94.7	27.7	98.4	28.7	0.707
	ALB (4.0–5.5 g/dL)	36.2	8.24	33.5	5.16	36.3	6.04	0.720
	Serum creatinine (53.0–140 umol/L)	112.3	87.0	137.7	123.3	77.0	19.2	0.302
	Serum β2 microglobulin (3.5–5.5 mg/L)	5.4	3.6	5.7	4.5	4.0	1.0	0.741
	D–S stage 1 2 and 3	4 60		2 26		0 5		0.829
	ISS I II and III	13 46		6 20		1 4		0.987
Nonspecific inflammatory markers (*n* = 115)	Platelet count (100–300 × 10^9^/L)	160.4	65.3	174.3	97.6	129.3	66.3	0.322
	Lym count (1.1–3.2 × 10^9^/L)	1.6	0.8	1.5	0.9	1.3	0.7	0.603
	Mono cout (0.1–0.6 × 10^9^/L)	0.3	0.2	0.3	0.2	0.4	0.2	0.760
	Lym/mono ratio	6.58	5.20	5.35	2.90	4.71	2.53	0.397
	Neu counts (1.8–6.3 × 10^9^/L)	3.9	2.4	3.7	2.0	2.7	1.1	0.435
	IL-6 (0–7.00 pg/mL)	14.4	13.7	7.4	4.2	58.0	19.8	0.006
	CRP (< 5 mg/L)	9.17	9.13	4.15	2.88	60.80	31.44	0.000
	PCT (< 0.046 ng/mL)	0.10	0,09	0.04	0.01	0.28	0.10	0.006
	LDH (110–220 IU/L)	179.2	116.1	180.1	69.6	209.8	43.27	0.743
	ESR (< 21 mm/h)	46.3	31.2	69.0	18.8	–	–	0.148
	Ferritin (24–336 ng/mL)	408.4	340.4	180.0	22.9	–	–	0.487
	BUN (3.38–8.57 mmol/L)	7.0	4.3	8.7	7.8	7.5	7.1	0.398
	Cys-c (0.51–1.09 mg/L)	1.53	0.91	2.12	1.89	2.1	2.0	0.087
	UA (240–490 umol/L)	411.7	175.2	352.6	151.1	354.0	122.0	0.211
	Triglyceride (0.29–1.83 mmol/L)	1.7	1.1	1.5	0.66	1.3	0.5	0.543
	Cholesterol (2.8–5.7 mmol/L)	3.8	1.3	3.8	1.3	3.6	1.0	0.857
	HDL-C (> 0.9 mmol/L)	1.1	0.38	1.1	0.39	1.3	0.72	0.387
	LDL-C (< 4.0 mmol/L)	2.2	0.93	2.2	1.03	1.4	0.35	0.148
	Blood glucose (3.9–5.9 mmol/L)	5.6	1.1	6.1	2.1	6.1	1.6	0.180
	EPO (3.7–29.5 mIU/mL)	93.9	73.4	17.2	4.5	–	–	0.304
Immune status indexes (*n* = 115)	ALG (2.0–4.0 g/dL)	47.4	23.5	50.5	24.7	48.1	14.4	0.820
	Normal Polyclonal Ig (g/dL)	28.3	9.17	37.0	25.2	35.5	5.6	0.078
	C3 (0.785–1.52g/L)	0.84	0.39	0.94	0.11	0.70	–	0.802
	C4 (0.145–0.36g/L)	0.19	0.09	0.13	0.12	0.21	–	0.603
	Properdin B (190–500 mg/L)	229.4	222.7	300.3	51.2	–	–	0.865
	CD3 (0.669–0.831)	0.62	0.12	0.66	0.04	–	–	0.611
	CD4 (0.3319–0.4785)	0.35	0.12	0.21	0.19	–	–	0.109
	CD8 (0.204–0.347)	0.25	0.08	0.33	0.12	–	–	0.195
	CD4/CD8 (0.97–2.31)	1.61	0.87	0.72	0.83	–	–	0.134
Bone disease indexes (*n* = 115)	Calcium (2.1–2.7 mmol/L)	2.23	0.32	2.09	0.43	2.33	0.40	0.136
	Inorganic Phosphorus (0.81–1.45 mmol/L)	1.16	0.35	1.30	0.46	1.5	0.7	0.088
	Magnesium (0.67–1.04 mmol/L)	0.87	0.30	0.83	0.30	0.87	0.14	0.861
	ALP (51–160IU/L)	82.97	60.57	78.18	37.77	69.83	29.43	0.801
	B-ALP (11.4–24.6 ug/L)	17.1	5.5	13.2	5.4	–	–	0.170
	X radiograph Osteoporosis Bone destruction Bone fracture	9 19 12		7 8 7		0 1 0		0.705
	BM biopsy Having myelofibrosis Having nomyelofirbosis	1 9		2 5		– –		0.36
Median time-before developing DR (months) (*n* = 115)		–		5 (1–32)		12.5 (3–27)		
Estimated OS (month) (*n* = 115)	80.1 (74.2–87.0)	84.1 (80.5–87.8)		80.5 (69.6–91.3)		42.6 (31.1–54.0)		0.001
Front-line therapy estimated OS (months)	80.22 (72.46–87.98)	83.167 (77.73–88.60)		72.00(63.998–80.002)		– –		0.761

**Table 3:** The relationship between the internal environment and treatment efficacy of thalidomide for MM

	Index	Response group (*n* = 18)		De novo DR (*n* = 40)		Acquired DR (*n* = 9)		*p*
		Mean	SD	Mean	SD	Mean	SD	
Age (year) (*N* = 67)	Median age and range (years) ≥ 65 < 65	60.5 (42–82) 6 12		63.0 (30–80) 16 24		67.0 (51–71) 5 4		0.539
Treatment condition (*n* = 67)	Front-line therapy Second-line therapy	14 4		27 13		8 1		0.371
Patients' general information (*n* = 67)	Isotype of M protein Light-chain Intact Ig Nonsecretory Missing	0 16 1 1		4 33 2 1		2 6 0 1		0.378
	Proportion of M protein	0.25	0.18	0.32	0.19	0.26	0.26	0.558
	Serum total involved/uninvolved light chain ratio 2–5:1 ≤ 0.01 or ≥ 100 0.01< ratio < 2 and 5 < ratio < 100	3 2 11		5 6 22		2 2 4		0,879
	Hb (13.0–17.5 g/dL)	104.7	26.6	104.8	29.5	97.4	35.6	0.800
	ALB (4.0–5.5 g/dL)	35.6	7.8	35.4	8.3	39.0	11.4	0.593
	Serum creatinine (53.0–140 umol/L)	79.1	35.8	101.9	72.26	141.66	78.5	0.092
	Serum β2 microglobulin (3.5–5.5 mg/L)	3.7	1.90	4.0	2.79	5.67	3.10	0.241
	D-S stage 1 2 and 3	5 27		4 17		2 7		0.884
	ISS I II and III	4 10		12 17		2 5		0.648
Nonspecific inflammatory markers (*n* = 67)	Platelet count (100–300 × 10^9^/L)	162.7	98.7	155.0	75.5	130.0	63.5	0.642
	Lym count (1.1–3.2 × 10^9^/L)	1.38	0.49	1.58	0.73	1.12	0.29	0.144
	Mono cout (0.1–0.6 × 10^9^/L)	0.34	0.17	0.28	0.16	0.33	0.21	0.456
	Lym/mono ratio	4.46	2.11	7.36	7.00	4.92	3.09	0.206
	Neu counts (1.8–6.3 × 10^9^/L)	3.75	2.60	3.43	2.21	1.93	1.00	0.183
	IL-6 (0–7.00 pg/ml)	6.22	5.163	4.45	4.03	3.18	1.19	0.634
	CRP (< 5 mg/L)	0.05	0.03	0.08	0.03	0.15	–	0.073
	PCT (< 0.046 ng/mL)	161.8	49.1	172.9	53.2	215.5	77.2	0.201
	ESR (< 21 mm/h)	72.5	44.1	67.6	39.0	18.0	5.66	0.262
	Ferritin (24–336 ng/mL)	–	–	786.0	643.0	–	–	–
	BUN (3.38–8.57 mmol/L)	6.26	2.12	6.36	3.02	8.81	4.53	0.117
	Cys-c (0.51–1.09 mg/L)	1.13	0.41	1.41	0.77	1.71	0.73	0.128
	UA (240–490 umol/L)	343.9	124.0	335.0	125.33	465.7	197.2	0.065
	Triglyceride (0.29–1.83 mmol/L)	1.6	0.9	1.5	1.0	2.4	1.2	0.082
	Cholesterol (2.8–5.7 mmol/L)	4.0	1.60	3.7	1.31	4.2	1.9	0.699
	HDL-C (> 0.9 mmol/L)	1.2	0.4	1.2	0.6	2.3	1.40	0.591
	LDL-C (< 4.0 mmol/L)	2.1	0.9	2.2	1.1	2.3	1.4	0.778
	Blood glucose (3.9–5.9 mmol/L)	7.15	4.2	5.73	1.85	5.93	0.88	0.191
Immune status indexes (*n* = 67)	ALG (2.0–4.0 g/dL)	42.3	22.1	48.9	41.5	42.94	31.50	0.598
	Normal polyclonal Ig (g/dL)	25.6	4.70	31.0	9.9	31.9	–	0.304
	C3 (0.785–1.52 g/L)	0.84	0.27	0.76	0.19	0.98	–	0.578
	C4 (0.145–0.36 g/L)	0.19	0.07	0.18	0.13	0.21	–	0.946
	Properdin B (190–500 mg/L)	271.4	92.2	200.6	74.5	–	–	0.171
	CD3 (0.669–0.831)	0.62	0.14	0.64	0.13	0.67	0.11	0.920
	CD4(0.3319–0.4785)	0.34	0.13	0.31	0.15	0.32	0.04	0.912
	CD8 (0.204–0.347)	0.24	0.06	0.29	0.11	0.31	0.04	0.477
	CD4/CD8 (0.97–2.31)	1.59	0.82	1.43	1.30	1.03	0.02	0.815
Bone disease indexes	Calcium (2.1–2.7 mmol/L)	2.13	0.55	2.05	0.23	2.29	0.50	0.403
(*n* = 67)	Norganic Phosphorus (0.81–1.45 mmol/L)	1.04	0.23	1.25	0.64	1.22	0.25	0.396
	Magnesium (0.67–1.04 mmol/L)	0.81	0.08	0.87	0.21	0.88	0.08	0.367
	ALP (51–160 IU/L)	69.8	24.8	74.99	47.1	61.4	16.6	0.678
	B-ALP (11.4–24.6 ug/L)	16.0	8.78	15.0	10.0	8.92	1.40	0.666
	X radiograph Osteoporosis Bone destruction Bone fracture	1 6 3		4 4 8		0 1 0		0.318
Estimated median time-before developing DR (months)	15 (1–57)	–	–	15 (1–54)		29.5 (4–57)		–
Estimated OS (months)	84.3 (73.2–95.5)	Not valid		88.2 (77.4–99.9)		66.0 (62.6–72.4)		0.753

As shown in Table 4, among 68 cases with cytogenetic results in this cohort, a statistically significant difference of a 1q21 gain frequency was found between the response and *de novo* DR groups (RR:3.472, 95% CI:1.184–10.179, *p* = 0.034). Moreover, patients with a 1q21 gain in the *de nov*o DR groups were significantly associated with lower levels of serum cholesterol (*p* = 0.029) and LDL-C (*p* = 0.024) (shown in Supplementary Table S1).

**Table 4:** The role of genetic abnormalities in predicting DR for MM in the new agent therapy era

		high-risk cytogenetics	1q gain	1p deletion	13p Deletion	TP53 deletion	t (4:14)	t (11;14)	t (14;16)
All (n = 68)	Response (n = 37) De novo (n = 24) Acquired (n = 7) p	13 15 4 0.096	12 15 4 0.057	0 1 1 0.111	12 9 4 0.460	1 1 1 0.391	2 2 0 0.700	0 1 2 0.003	0 1 0 0.394

### Exosomes and exosomal RNAs isolated from culture media and peripheral blood of MM

**Figure 1:**
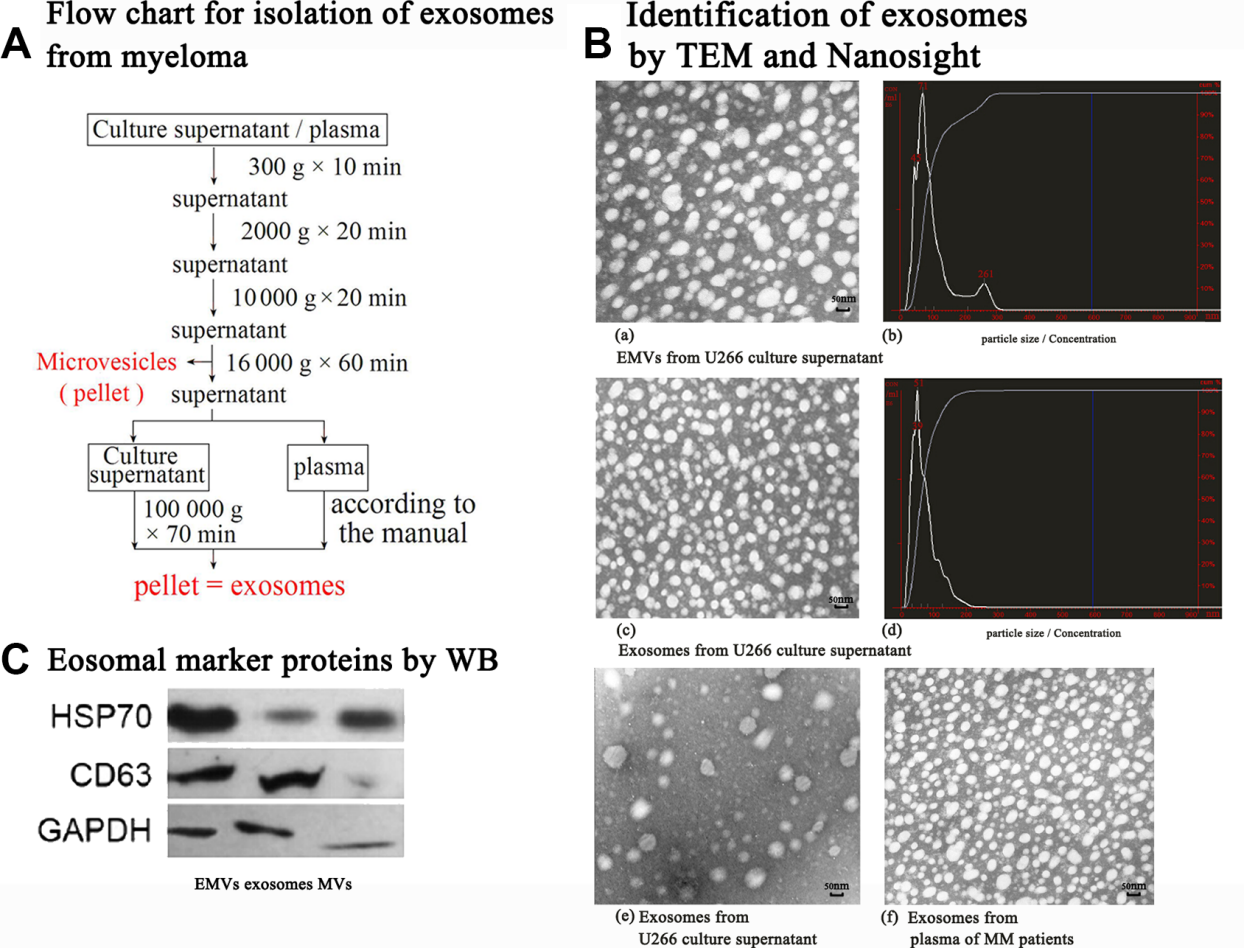
Isolation and validaton of exosomes from both U266 cell line and MM patients MM-derived exosomes were isolated and purified as depicted in section A. In section B, presence of MVs and exosomes: Both MVs and exosomes were present by TEM (**A**) and Nanosight (**B**) following centrifugation of U266 culture supernatant without removal of MVs by 16 000 g × 60 min; Exosomes were present by TEM (**C**) and Nanosight (**D**) following centrifugation of U266 culture supernatant with removal of MVs by 16 000 g × 60 min. Processed plasma samples from MM patients based on the protocol in section A, exosomes derived from MM quantitatively predominated (**F**) than those from the healthy control (**E**). In section C, EMVs, exosomes and MVs were isolated from the culture supernatant of U266 cells, respectively. Exosomal marker proteins of HSP70 were detected for both exosomes and MVs, while CD63 was mainly observed in exosomes.

**Figure 2:**
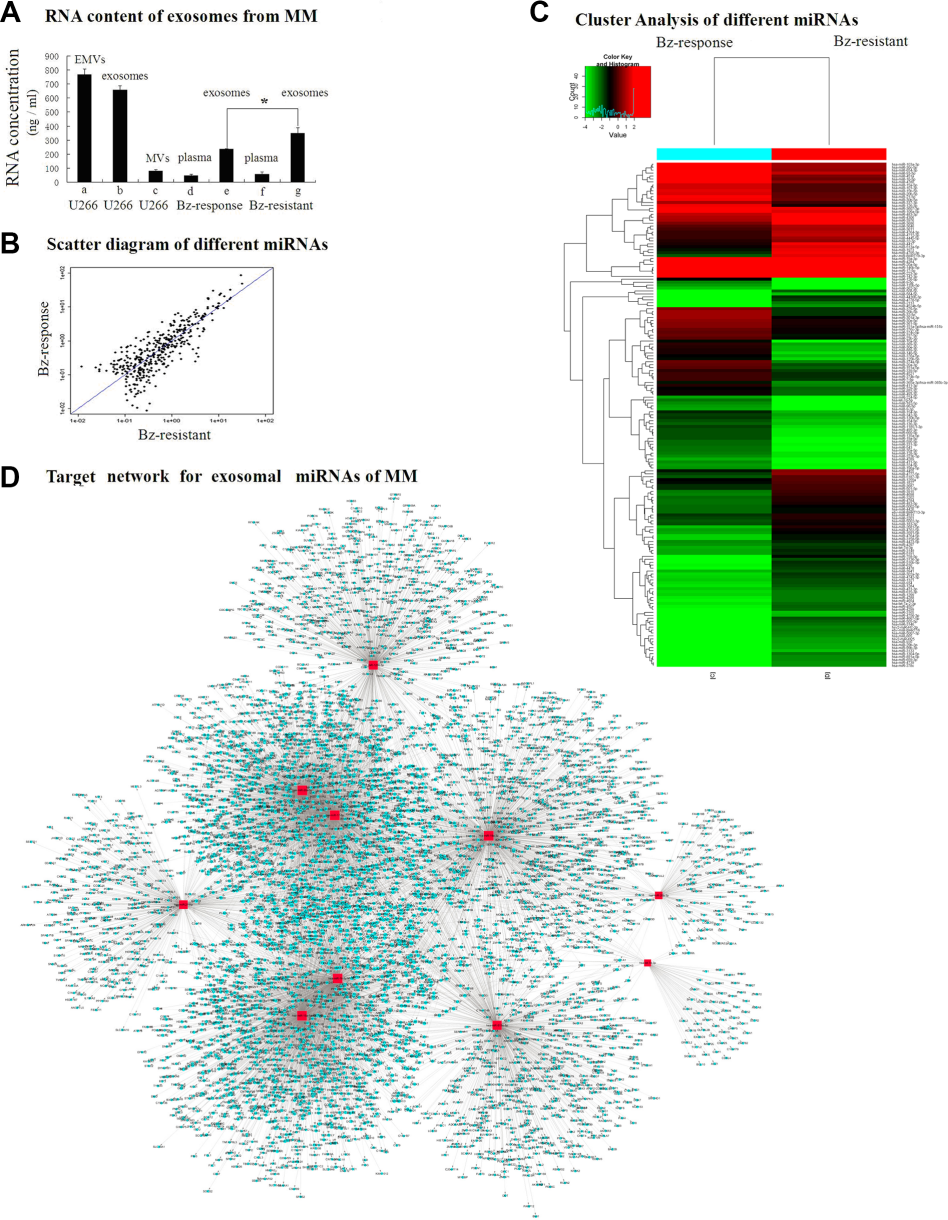
The differential expression of exosome-associated miRNAs in the subgroups of MM Exosomal RNAs were detected from both the culture supernatant of U266 cells and the plasma samples from MM patients (**A**). Significant differences (* < 0.05) of exosomal RNA content were observed between the Bz-response group (E) and Bz-resistant group (**G**). Exosomal RNA content of both groups was much higher than the content of circulating RNAs (**D** and **F**) extracted using the miRNeasy Serum/Plasma Kit (Qiagen). In sections (**B** and **C**) compared to the Bz-response group, 83 miRNAs were expressed at higher levels and 88 miRNAs were expressed at lower levels in the Bz-resistant group among 3180 miRNAs on the microarray. In section D, a miRNA-RNA synergistic network was constructed with miR-17-5p, miR-20a-5p and miR-15a-5p, miR-16-5p exhibiting more synergism in the Bz-resistant mechanism of MM.

As shown in Figure 1B, TEM images and NanoSight NS300 revealed the presence of nano-sized, round EMVs, MVs and cup-shaped exosomes isolated from the U-266 cell culture media, serum of MM patients and healthy control. The purified exosomes of less than 100 nm in diameter, which are equipped with exosomal marker proteins CD63 and HSP70 (Figure 1C), were more abundant in the serum of MM patients than in healthy control. As shown in Figure 2A, significant differences of exosomal RNA content were observed between the Bz-resistant group and the Bz-response group (350.17 ± 37.55 ng/μl *vs* 235.43 ± 3.91 ng/μl, *p* = 0.033) by Nanodrop analysis and the exosomal RNA concentrations of both groups were obviously higher than 78.21 ± 45.51 ng/μl of the healthy control. Besides, we also isolated circulating RNAs in plasma of patients in the Bz-resistant group and the Bz-response group (Figure 2A–2D and Figure 2A–2F). It showed that the exosomal RNA concentrations of both groups were significantly higher than the circulating RNA concentrations, respectively (data not shown).

### The exosome-associated miRNA expression patterns for predicting Bz-resistance in MM

Among 3180 miRNAs detected on a microarray, profiling data analysis predicted that 83 miRNAs were expressed at higher levels and 88 miRNAs were expressed at lower levels in Bz-resistant group (Figure 2B), and more than 90% of these miRNAs had novel functions. After result confirmation by RT-PCR to remove false positives, we listed the top 10 miRNAs exhibiting the largest changes, overlapped with the top miRNAs based on literature, namely, miR-513a-5p, miR-20b-3p, let-7d-3p were up-regulated and miR-125b-5p, miR-19a-3p, miR-21-5p, miR-20a-5p, miR-17-5p, miR-15a-5p, miR-16-5p were down-regulated. These differentially expressed miRNA families play key roles in post-transcriptional regulation by influencing transcription co-factors, the MAP kinase pathway and ubiquitin conjugating enzyme activity (Figure 2C). Furthermore, a miRNA-RNA synergistic network was constructed to describe the role of multiple exosomal miRNAs in the DR mechanisms of complex post-transcriptional regulations in human MM, which were by several miRNAs rather than a single miRNA. We found that miR-17-5p, miR-20a-5p, miR-15a-5p and miR-16-5p exhibited higher synergistic effects (Figure 2D), indicating a functional complexity where a global central core of the post-transcriptional regulatory network is involved as a Bz-resistant mechanism of MM. Levels of miR-16-5p, miR-15a-5p, miR-20a-5p and miR-17-5p in the Bz-resistant group were 3.91, 1.83 and 2.96, 1.97 folds lower, respectively, than those in the Bz-response group.

## DISCUSSION

Early predication of DR has been playing an increasingly important role in holistic treatment for patients with MM, considering that nearly all patients eventually relapse and refractory MM (RRMM) with multidrug resistance is observed even with the use of novel treatment agents. Moreover, almost 20–30% of MM patients have innate DR to Bz [12, 13]. The present study showed that Bz and thalidomide were mainly used as front-line therapies (67.2%, 71.3%, respectively) and lenalidomide as a salvage therapy (72.7%), differing from the management guideline for multiple myeloma (Version4, 2015) published by the National Comprehensive Cancer Network (NCCN). To some extent, this may be associated with an overwhelming price advantage of thalidomide over lenalidomide in the Chinese market and this may generally represent the current MM treatment in China. In this study we explored the potential factors responsible for the occurrence of Bz and thalidomide DR.

Compared with the Bz-response groups, risk predictors for an increased likelihood of acquired resistance were in descending order, namely, abnormal total serum light chain ratio (≤ 0.01 or ≥ 100), higher CRP level (≥ 20 mg/L), and the second-line usage. First, although the free light chain (FLC) ratio has been increasingly used for monitoring MGUS evolving to MM and early evaluation of Bz response, the test of FLC ratio has not been widely available in the majority of Chinese myeloma centers [14, 15]. Under such circumstances, the clinical role of the total light chain ratio in Bz resistance cannot be ignored. The total serum light chain detection in this analysis was consistent with results from a published retrospective study that speculated that reaching a normal total light chain ratio may be sufficient to maintain a stable phase of the disease and confer prolonged event free survival (EFS) [16]. Second, a high level of CRP is known to contribute to a high tumor burden, extensive complications and poor prognosis in MM [17, 18]. In contrast, CRP may protect myeloma cells from dexamethasone and melphalan-induced apoptosis *in vitro* and *in vivo* [19]. A shorter time to progression was correlated with an elevated CRP level during the use of Bz-containing therapy, which suggested that a high level of CRP was involved in the rapid progress due to resistance to Bz [20]. Meanwhile, according to the up-to-date data [21, 22], CRP elevation was one of the potential side effects of Bz, especially for male patients older than 60 years old using the drug less than 1 month, but the mechanisms of DR remain largely unknown. Thereby, monitoring CRP changes is essential during Bz usage and lowering CRP to the baseline level may offer a potential way to alleviate the risk of Bz acquired resistance. In reference to the inferiority of the second-line usage of Bz for relapsed disease, the phase III VISTA trial and the phase II RETRIEVE study analyzed the efficacy of Bz retreatment after the initial response to Bz, with a response rate (≥ PR) in the range of 21%–50% [23, 24]. In contrast, Bz was approved by the FDA for treating relapsed MM patients in 2003, this was due, to a large extent, to data from 202 cases for whom previous therapies did not work, including traditional the chemotherapy of thalidomide or hematopoietic stem cell transplantation (HSCT) from 14 centers [25]. The response rate of second-line usage of Bz for RRMM was 59% [26–29]. In this study, the drug resistance rate to second-line usage of Bz was as high as 54.5% (*n* = 18) for the whole RRMM and 50% for the RRMM previously obtaining the Bz treatment. The clonal evolution theory indicates that there are emerging dominant clones that may now be drug resistance to the same drug [21]. Until now, for RRMM, whether the ineffective agent used previously could be reused for MM has given rise to a heated debate and awaits further investigation.

It has not been possible to assess the impact of the *in vivo* environment in the subgroup of patients with MM presenting *de novo* resistance to Bz, as well as both primary and acquired DR to thalidomide. Prospective and larger clinical trials are clearly needed to resolve this issue. The use of precise medicine would be a promising model providing tailored therapies to individual patients especially when the *in vivo* microenvironment influences the pathological processes via the interaction with MM cancerous components that varies from patient to patient. In addition, no correlation between DR and immune status indicators (bone metabolism and bone disease indicators) has been found in the multivariate analysis, which may be partly explained by the limited number of patients in our cohort and other factors should also be taken into considerations, namely, 1) the sensitivity of the measurements, for example, plain radiographs rather than MRI may show false negative results for myeloma-induced bone lesions, and 2) dynamic parameter tracking for particular individuals.

The number of samples with genetic information has been fairly limited. Among 63 cases with FISH results in the cohort, the gain of 1q21 has been implicated as a risk factor for conferring *de novo* DR of the new agents. In line with this possibility, increasing evidence has suggested that the 1q21 gain accounted for DR and poor OS [30, 31]. The 1q21 gain is also correlated with lower levels of serum cholesterol and LDL-C. In fact, metabolic pathways in multiple myeloma are essential for the regulation of DR [32], but based on genetic evidence, chromosome 1q21 was associated with type 2 diabetes susceptibility in Hong Kong Chinese [33].

Since there has not been a practical model from the routine workup of MM to effectively predict DR, we aimed to construct a model based on the differential expression of exosomal miRNAs. Given that Bz is the most widely used new drug for MM, exosome-associated miRNA panels, reflecting the crosstalk between MM cells and the *in* vivo environment, were explored in this study by comparing the Bz-resistance and Bz-response groups. Although *in vivo* tracking of the exosomes in humans is challenging, a conclusion is that the majority of the exosomes come from myeloma cells because the significant elevation of the total quantification of RNA when comparing both the myeloma subgroups to the healthy control and the resistant group to the response group. The microarray profiling found four exosomal miRNAs (miR-16, miR-15a, miR-20a and miR-17) are at the core of the miRNA-RNA regulatory signaling network predicted by integrating network analysis and Gene Ontology and were confirmed by quantitative real-time PCR. Our previous work and other findings suggest that down-regulation of miR-15a and miR-16 of primary MM cells as well as in MM cell lines could contribute to the DR and progression by the modulation of the bone marrow microenvironment [34, 35]. In addition, miR-20a and miR-17 are also involved in the tumorigenicity of MM [36]. Although miR-15a and miR-16 are located on 13q13.4 and miR-17-92 cluster of chromosome 13q31.3, their expression is independent of the loss of chromosome 13, which is observed in almost half of MM patients and resulted in poor prognosis. Further prospective studies on a larger cohort are required to clarify the potential role of specific exosomal miRNAs, namely, miR-15a and miR-16 with the putative BCL2 target and miR-20a and miR-17 with the putative Myc target.

Our study has the important implication of using serum exosomal miRNAs as drug resistance biomarkers for MM. First, cell-free circulating RNAs have opened a window to assess global alteration of the *in vivo* disease and an extensive list of circulating RNA-based biomarker candidates have been reported in the recent years [37, 38]. Significantly, in this study from the equivalent blood sample of the same patients, obviously higher concentrations of exosomal RNAs than circulating RNAs were found, suggesting that more effort is necessary to make a comprehensive assessment of the potential roles of exosomal RNAs in human disease. Second, unlike monogenic disorders such as chronic myeloid leukemia with the formation of the BCR-ABL fusion gene [39], MM is a complex polygenic trait shaped by multiple and translocation genetic anomalies, thus leading to a more obvious disease heterogeneity. The advances in systems biology and the development of new molecular tools in the “omics” science, including genomics, Rnomics, miRNomics, proteomics and epigenomics, have made it possible to reveal new findings in relation to DR in MM patients. The expression panel of miRNAs that are highly stable in blood and involved in a complex, multi-faceted network of regulatory interactions suggest that miRNAs may be the preferred option for circulating biomarker for MM in routine clinical practice since mRNAs and proteins are unstable molecules, in constant alteration and dynamic expression of mRNAs and proteins are frequently correlated with the events of each episode taking place within extended periods of time. Although DNAs are biologically stable, their mutations exert a great deal of diversity due to the complexity of assembled genomic clones and biological subtypes among individual MM cases. Our results still warrant further investigations because of the limitation of number of miRNAs microarray in each group and outside of Bz. Moreover, large multi-center studies are needed to explore the more precise exosome-associated microRNA expression models accompanying the development of novel agents.

## MATERIALS AND METHODS

### Patients

From January 2013 to December 2014, all the hospitalized MM patients in our tertiary hematology center, who received novel agents-based therapies, namely, bortezomib (Bz), thalidomide or lenalidomide, and did not participate in any clinical trials during the corresponding period, were enrolled in this study. This study was performed in accordance with the 1996 Declaration of Helsinki and approved by the ethics committees of West China Hospital, Sichuan University. The diagnosis of MM was established using the International Myeloma Working Group 2003 diagnostic criteria [40]. The cut-off date for follow-up was March 31st, 2015. The median follow-up was 23 months with a median follow-up of 23 months, and 38 patients (19.1%) were lost from follow-up sessions.

### Study design and treatment

The overall treatment for patients with MM was divided into three phases: induction, consolidation and maintenance. Thalidomide-base therapy of MPT regimen specifically included melphalan orally 0.25 mg/kg on days 1–4, prednisone orally 1 mg/kg on days 1–4 and thalidomide orally 50–200 mg/day, with over 4–6 weeks as per cycle. Bz-based therapy of BD regimen specifically included Bz intravenously or subcutaneously 1.3 mg/m^2^ on days 1, 4, 8, 11 and dexamethasone orally or intravenously 20 mg/day on days 1–2, 4–5, 8–9, 11–12 with over 21-days per cycle. Lenalidomide-based therapy of Rd regimen specifically included lenalidomide orally 25 mg/day on days 1–21 and dexamethasone orally 20 mg on days 1, 8, 15 and 22 with over 28-days per cycle. After at least 4 cycles of induction treatment, patients with partial remission or better response underwent consolidation therapy, either autologous stem cell transplant (ASCT) or chemotherapy with the initial regimens, according to their intent and performance status. Subsequently, the patients with good tolerance would be treated with thalidomide (100–150 mg/day) for 1 year for maintenance, if the drug was well tolerated. The role of the *in vivo* environment was analyzed as contributors to DR, while the data based on the routine workup for MM were collected into 5 aspects as follows: (1) patients' general information; (2) immune status indicators; (3) bone disease indicators; (4) nonspecific inflammatory markers; (5) genetic profile. More details are found in the Tables.

DR was defined according to the criteria of the 19th annual meeting of European Hematology Association. Briefly, the patients were divided into two groups based on therapeutic outcome: the response group and resistant group. The latter were further divided into the *de novo* drug resistant group (*de novo* DR) and the acquired drug resistant group (acquired DR). The *de novo* DR consisted of the patients who failed to achieve minimal remission (MR) or experienced progressive disease (PD) within 60 days when receiving the novel agent-based therapy for the first time, while the acquired DR consisted of those who developed DR when receiving the novel agent-based therapy and had previously been treated with the drug. The response group represented the patients who acquired partial remission (PR) or complete remission (CR) no matter the first or second line application of the novel agent-based regimen. Duration of response was defined as the time from the date of the first response to the date of PD or death due to PD. Survival time was defined as the time from diagnosis to the last follow-up or death.

### Cell lines, cell culture and blood sample collection

The human multiple myeloma U266 cell line was purchased from American Type Culture Collection (ATCC) and cultured in RPMI 1640 containing 10% fetal bovine serum (FBS) at 37°C in 5% CO_2_. For exosome isolation, the U-266 was prepared with 10% SBI EXO-FBS-50A-1 Exosome-depleted FBS media supplement. Blood samples of the patients who would receive Bz-based regimen were collected into EDTA-coated tubes and in the same day processed under a protocol approved by the Institutional Review Board in Sichuan University, West China Hospital. Blood samples of the patients who preferred to receive the novel drug-based regimen were collected with written informed consent.

### Exosome isolation and identification

Extracellular membrane microvesicles (EMVs) are circulating fragments of membrane, including exosomes, which are released from the endosomal compartments with diameters of 30 to 100 nm and microvesicles (MVs), which are shedding from the surface membranes of most cell types with diameters of 50 to 2,000 nm. Until now, even differential ultracentrifugation, the gold standard method for separating and purifying EMVs, is incapable of efficiently distinguishing between exosomes and MVs. Alternatively, based on the principle of aqueous gradient solubility differences between various lipids and nanoparticles, commercial kits are able to capture EMVs and then use either 0.2-micrometer (μm) pore size filters or artificially synthesized molecular sieves to enrich exosomes. In this study, we isolated and purified exosomes from both U266 cell line and MM samples, as depicted in Figure 1A. Then, the size and morphology of EMVs were observed by transmission electron microscope (TEM) (hitachi H-600 Japan) after negatively staining by 2% uranyl acetase. The size distribution of EMVs was traced by NanoSight NS300 (Malvern company, Great Britain) following manufacture protocols. Protein lysates of EMVs were assessed by Coomassie staining and western blotting for identification the expression of exosomal protein marker such as heat shock protein (Hsp70), CD63 and myeloma membrane-associated CD138.

### RNA isolation and microRNA array profiling

Serum exosomal samples of 3 subjects were selected and harvested, respectively, from Bz-response and Bz-resistant groups. To extract circulating RNA from plasma samples, the miRNeasy Serum/Plasma Kit (Qiagen, Germany) was used according to the manufacturer's instructions. Total RNA from cells or exosomes harvested from serum was isolated using TRIzol (Invitrogen, Carlsbad, CA). Quantity and quality of RNA was evaluated by nanodrop spectrophotometer (ND-1000, Nanodrop Technologies). An equal amount of 150 ng RNA from the 3 subjects of the same group was mixed to minimize differences among subjects within a group. Total RNA from both groups was labeled with Hy3^™^ fluorescent using the miRCURY^™^ Hy3^™^/Hy5^™^ Power labeling kit (Exiqon, Vedbaek, Denmark) following the procedure described by the manufacturer. The Hy3^™^-labeled samples were mixed and hybridized to the miRCURY^™^ LNA Array version 7th Generation (Exiqon), covering all human, mouse and rat miRNAs annotated in miRBase 18.0, as well as all viral microRNAs related to these species. In addition, this array contains capture probes for 25 miRPlus^™^ human miRNAs. The hybridization was performed according to the miRCURY^™^ LNA array manual. Then, the slides were scanned using the Axon GenePix 4000B microarray scanner (Axon Instruments, Foster City, CA), and scanned images were then imported into GenePix Pro 6.0 software (Axon) for grid alignment and data extraction. Replicated miRNAs were averaged and miRNAs that intensities > = 30 in all samples were chosen for calculating normalization factor. Expressed data were normalized using the Median normalization. After normalization, differentially expressed miRNAs were identified through Fold Change filtering. Hierarchical clustering was performed using MEV software (v4.6, TIGR). MicroRNA array experiments were performed by the Shanghai KangChen Bio-tech Company, Shanghai, China. Real-time PCR was used for confirmation. Differentially expressed miRNAs were identified through volcano plot screening. Cluster analysis was carried out by hierarchical clustering. Finally, a fold change analysis was performed by calculating the ratio between the two groups with a cut-off value of 2-fold changes. The mechanism of miRNA differentially expressed between groups were further explored by Ontology (GO) classification analyses through evaluating the genes affected by the upregulated and downregulated miRNAs, and Kyoto Encyclopedia of Genes and Genomes (KEGG) for pathway analysis were also employed to estimate the functions and pathways of miRNAs target genes.

### Statistical analysis

Results were analyzed using SPSS version 13.00 software. For categorical variables, statistical data were described as frequency counts and percentages; for continuous variables, average, medians and ranges were adopted. Comparison of ratio and constituent ratio were using chi-square test. Median and average were computed by using independent samples *T*-test or one-way ANOVA. Kaplan-Meier method was used to estimate duration of response and OS, and the differences between groups were computed using stratified log-rank tests. All statistical tests were 2-sided, and *P* < 0.05 was considered statistically significant for all comparisons and confidence intervals refer to 95% boundaries.

## SUPPLEMENTARY MATERIALS TABLE




