# Supplementary material for: Potential role of exosome-associated microRNA panels and in vivo environment to predict drug resistance for patients with multiple myeloma
Source: Oncotarget. 2016 Apr 26;7(21):30876–91. doi: 10.18632/oncotarget.9021 (PMC5058725; doi:10.18632/oncotarget.9021)
Supplement: Supplementary file 1 [file oncotarget-07-30876-s001.pdf]

Potential role of exosome-associated microRNA panels and *in vivo* environment to predict drug resistance for patients with multiple myeloma

Supplementary Materials

Supplementary Table S1: The relationship between *in vivo* environment and genetic abnormalities to predict DR for MM in the new agent era. See Supplementary_Table_S1
