# Supplementary material for: Potential role of exosome-associated microRNA panels and in vivo environment to predict drug resistance for patients with multiple myeloma
Source: Oncotarget. 2016 Apr 26;7(21):30876–91. doi: 10.18632/oncotarget.9021 (PMC5058725; doi:10.18632/oncotarget.9021)
Supplement: Supplementary file 2 [file oncotarget-07-30876-s002.docx]

**Supplementary Table S1: The relationship between *in vivo* environment and genetic abnormalities to predict DR for MM in the new agent era.**

| **Index** | **1q21** | | ***P*** |
| --- | --- | --- | --- |
|  | **(+)**  **Mean SD** | **(–)**  **Mean SD** |  |
| Hb  The response group  The *de novo* group | 86.09 24.80  92.25 22.63 | 103.50 23.24  112.25 35.75 | 0.101 |
| PLT  The response group  The *de novo* group | 151.91 51.24  135.33 56.46 | 172.25 52.38  187.87 106.14 | 0.235 |
| Neu  The response group  The *de novo* group | 3.30 1.53  3.46 1.83 | 4.05 3.13  4.25 2.13 | 0.771 |
| Lym  The response group  The *de novo* group | 1.36 0.64  1.56 0.72 | 1.69 0.64  1.88 0.81 | 0.400 |
| Mono  The response group  The *de novo* group | 0.31 0.17  0.27 0.15 | 0.33 0.17  0.43 0.26 | 0.446 |
| Lym/mono  The response group  The *de novo* group | 6.46 4.44  5.62 2.99 | 5.80 2.87  5.23 2.62 | 0.920 |
| ALB  The response group  The *de novo* group | 34.86 9.32  34.67 11.76 | 38.58 6.89  28.70 12.87 | 0.306 |
| GLB  The response group  The *de novo* group | 57.65 26.43  44.20 19.75 | 43.73 23.00  44.77 27.07 | 0.388 |
| Blood glucose  The response group  The *de novo* group | 6.30 2.09  6.17 2.24 | 6.02 2.48  5.41 0.63 | 0.830 |
| BUN  The response group  The *deo novo* group | 8.05 7.00  7.67 6.79 | 5.71 1.58  4.91 0.84 | 0.285 |
| CREA  The response group  The *de novo* group | 100.01 69.47  72.39 13.00 | 81.15 31.92  81.61 20.86 | 0.425 |
| Cys-c  The response group  The *de novo* group | 1.60 1.37  1.69 1.56 | 1.20 0.31  1.31 0.54 | 0.478 |
| UA  The response group  The *de novo* group | 399.33 206.88  335.04 148.45 | 403.35 100.70  344.10 149.54 | 0.513 |
| Triglyceride  The response group  The *de novo* group | 1.50 1.02  1.41 0.73 | 1.77 0.79  1.93 1.17 | 0.522 |
| Cholesterol  The response group  The *de novo* group | 3.09 0.97  3.95 1.19 | 4.20 0.90  4.40 1.53 | 0.029 |
| HDL-C  The response group  The *do novo* group | 0.97 0.33  1.34 0.46 | 1.11 0.27  1.16 0.28 | 0.082 |
| LDL-C  The response group  The *denovo* group | 1.65 0.66  2.12 0.85 | 2.52 0.74  2.56 1.13 | 0.024 |
| ALP  The response group  The *de novo* group | 65.08 25.09  79.33 31.22 | 69.34 27.98  64.11 35.90 | 0.596 |
| LDH  The response group  The *de novo* group | 138.0 45.00  171.64 40.29 | 161.67 32.07  193.88 70.21 | 0.060 |
| Calcium  The response group  The *de novo* group | 2.19 0.45  2.05 0.36 | 2.26 0.20  1.91 0.55 | 0.140 |
| Magnesium  The response group  The *de novo* group | 0.84 0.11  1.04 0.48 | 0.83 0.08  0.75 0.10 | 0.050 |
| Inorganic Phosphorus  The response group  The *de novo* group | 1.20 0.39  1.29 0.60 | 1.10 0.26  1.29 0.42 | 0.547 |
| β2  The response group  The *de novo* group | 4.93 3.18  3.38 1.87 | 4.74 2.72  5.81 4.66 | 0.418 |
| Proportion of M protein  The response group  The *de novo* group | 0.40 0.23  0.36 0.17 | 0.26 0.20  0.40 0.20 | 0.262 |
| Normal Polyclonal Ig  The response group  The *de novo* group | 30.15 10.09  26.13 10.01 | 24.78 12.61  27.85 19.70 | 0.768 |
| IL-6  The response group  The *de novo* group | 6.31 3.45  2.38 0.40 | 37.92 31,.77  6.70 4.01 | 0.099 |
| CRP  The response group  The *de novo* group | 5.52 0.48  2.21 1.72 | 15.22 15.00  4.50 3.69 | 0.190 |
| PCT  The response group  The *de novo* group | 0.12 0.04  0.04 0.01 | 0.09 0.08  0.05 0.04 | 0.348 |
| ESR  The response group  The *de novo* group | 68.50 72.83  61.00 49.00 | 58.33 32.71  57.00 39.95 | 0.991 |
| CD3  The response group  The *de novo* group | 0.70 0.09  0.75 —- | 0.59 0.14  - | 0.288 |
| CD4  The response group  The *de novo* group | 0.36 0.08  0.40 —- | 0.29 0.05  —— | 0.268 |
| CD8  The response group  The *de novo* group | 0.31 0.03  0.31 —- | 0.24 0.13  - | 0.639 |
| CD4/CD8  The response group  The *de novo* group | 1.14 0.16  - | 1.47 1.00  - | 0.587 |
| Properdin B  The response group  The *de novo* group | 122.0–  241.67 36.90 | 325.33 112.43  269.00 125.87 | 0.135 |
| C3  The response group  The *de novo* group | 0.59 0.13  0.84 0.27 | 0.93 0.22  0.86 0.26 | 0.270 |
| C4  The response group  The *de novo* group | 0.088 0.088  0.13 0.12 | 0.20 0.07  0.14 0.17 | 0.477 |
| B-ALP  The response group  The *de novo* group | 24.56–  15.16 6.79 | 15.14 3.77  15.67 11.92 | 0.658 |
| Serum total involved/uninvolved light chain ratio  ≤ 0.01 or ≥ 100  0.01< ration< 100 | 3  18 | 0  27 | 0.077 |
| Front-line therapy  Second-line therapy | 19  9 | 27  6 | 0.244 |
| CRP≥ 20 mg/L  CRP< 20 mg/L | 1  9 | 4  12 | 0.617 |
